# Plasmon-Activated Water Reduces Amyloid Burden and Improves Memory in Animals with Alzheimer’s Disease

**DOI:** 10.1038/s41598-019-49731-8

**Published:** 2019-09-13

**Authors:** Chia-Hsiung Cheng, Kun-Ju Lin, Chien-Tai Hong, Dean Wu, Hung-Ming Chang, Cheng-Huan Liu, Ing-Tsung Hsiao, Chih-Ping Yang, Yu-Chuan Liu, Chaur-Jong Hu

**Affiliations:** 1 Department of Biochemistry and Molecular Cell Biology, School of Medicine, College of Medicine, Taipei Medical University, 250 Wuxing St., Taipei, 11031 Taiwan; 2 Department of Nuclear Medicine and Molecular Imaging Center, Linkou Chang Gung Memorial Hospital, No.5, Fuxing St., Taoyuan City, 333 Taiwan; 3 Healthy Aging Research Center and Department of Medical Imaging and Radiological Sciences, College of Medicine, Chang Gung University, 259 Wenhua 1st Rd., Taoyuan City, 33302 Taiwan; 4 Department of Neurology, School of Medicine, College of Medicine, Taipei Medical University, 250 Wuxing St., Taipei, 11031 Taiwan; 5 Department of Neurology and Dementia Center, Shuang Ho Hospital, Taipei Medical University, 291 Jhongjheng Rd., Jhonghe, New Taipei City, 23561 Taiwan; 6 Department of Anatomy and Cell Biology, School of Medicine, College of Medicine, Taipei Medical University, 250 Wuxing St., Taipei, 11031 Taiwan; 7 Science Department of Physiology, McGill University, 3655 Promenade Sir William Osler, Montreal, Quebec, H3G 1Y6 Canada; 8 PhD Program of Neural Regenerative Medicine, College of Medical Science and Technology, Taipei Medical University, 250 Wuxing St., Taipei, 11031 Taiwan

**Keywords:** Hippocampus, Neurodegenerative diseases

## Abstract

With the great extension of the human lifespan in recent times, many aging diseases have inevitably followed. Dementia is one of the most-commom neurodegenerative aging diseases, in which inflammation-related Alzheimer’s disease (AD) is the most prevalent cause of dementia. Amyloid accumulation in the brain, which occurs before any clinical presentations, might be the first and key step in the development of AD. However, many clinical trials have attempted to remove amyloid from brains of AD patients, but none has so far been successful. Negatively charged plasmon-activated water (PAW) is created by resonantly illuminated gold (Au) nanoparticles (NPs), which reduce the hydrogen-bonded (HB) structure of water. PAW was found to possess anti-oxidative and anti-inflammatory effects. Herein, we report on an innovative strategy to retard the progression of AD by the daily consumption of PAW instead of normal deionized (DI) water. APPswe/PS1dE9 transgenic mice were treated with PAW or DI water from the age of 5 months for the next 9 months. Encouragingly, compared to DI water-treated mice, mice treated with PAW presented better memory performance on a test of novel object recognition and had a significantly lower amyloid burden according to 18F-florbetapir amyloid-PET and phosphorylated (p)-tau burden according to Western blotting and immunohistochemistry measurements. There were no obvious side effects in PAW-treated mice. Collectively, our findings support that PAW was able to reduce the amyloid and p-tau burden and improve memory in an AD mouse model. However, the protein levels of molecules involved in amyloid metabolism and oligomeric amyloid did not change. We propose that the effects of PAW of reducing the amyloid burden and improving memory function cannot be attributed to synthesis/degradation of amyloid-βprotein but probably in preventing aggregation of amyloid-β proteins or other mechanisms, including anti-inflammation. Further applications of PAW in clinical trials to prevent the progression of AD are being designed.

## Introduction

The number of dementia patients is increasing worldwide. Along with aging in most populations, the treatment and care of dementia have become urgent public health issues for most countries^1,2^. Alzheimer’s disease (AD) is the most common cause of dementia, and AD is characterized by memory impairment followed by declines in many cognitive domains, including executive, visuospatial, and other functions, resulting in an inability to carry out activities of daily life. AD is a progressive disease, and during the disease course, patients usually present many kinds of behavioral psychological symptoms of dementia, such as confusion, hallucinations, agitation, and sleep disturbances. In severe and advanced stages of AD, patients are totally dependent or even bed-ridden^3,4^. AD not only affects patients but also creates huge burdens for caregivers, families, and society^5,6^. The real causes of AD are still unclear. Both extracellular senile plaques and intracellular neurofibril tangles are markers of AD pathology; therefore, amyloid and tau which are the main components, respectively, of senile plaques and neurofibril tangles, are considered to play important roles in the pathophysiology of AD^7,8^. β-Amyloid (Aβ) consisting of 40 or 42 amino acids is produced from the amyloid precursor protein (APP) after digestion by β-secretase and γ-secretase. Aβ, especially in its oligomeric form, is neurotoxic, and mutations of the *app*, presenilin 1 (*psen1*), or presenilin 2 (*psen2*), both of which are involved in γ-secretase activity, result in familial-type AD. Therefore, amyloid should be a key factor in AD^9^. Compelling evidence suggests that the progressive course of AD could initially be amyloid accumulation followed by tau accumulation, impairment of brain metabolism, atrophy of the medial temporal lobe, and the appearance of clinical manifestations^10^. The aging process, oxidative stress, and chronic inflammation may contribute to amyloid accumulation^11,12^. The accumulation of tau, especially phosphorylated (p)-tau, is actually more compatible with the clinical severity and progression of pathological findings in AD than is amyloid^7,13^. Many studies have tried to stop Aβ formation, including inhibitors of β-secretase or γ-secretase, or removing Aβ, such as with a vaccine, or increasing Aβ degradation, such as with neprilysin enhancers. However, no method has so far been successful^14^. Currently, it is basically only possible to treat the symptoms of AD. Only a few drugs are approved by the US Food and Drug Administration (FDA) for AD therapy^15^.

As reported by Ohsawa *et al*.^16^, hydrogen can act as a therapeutic antioxidant by selectively reducing cytotoxic oxygen radicals. As reported by Yoo *et al*.^17^, electromagnetized gold nanoparticles (AuNPs) can mediate direct lineage reprogramming into induced dopamine neurons *in vivo* for Parkinson’s disease therapy. On the other hand, plasmon-activated water (PAW) is an innovative invention that possesses numerous advantages compared to conventional deionized DI water^18^. By letting DI waterflow through supported gold nanoparticles (AuNPs) under resonant illumination, effective hot electron transfer breaks the hydrogen bonds and thus makes PAW more active than regular water in various chemical and physical reactions^18,19^. The resulting stable PAW exhibits distinct properties at room temperature, which significantly differ from those of untreated DI water, e.g., its ability to scavenge free hydroxyl and 2,2-diphenyl-1-picrylhydrazyl (DPPH) radicals and effectively reduce nitric oxide (NO) release from lipopolysaccharide-induced inflammatory cells. Moreover, the created PAW is energetic and more effective in solubility than DI water^18,20,21^. Energetic PAW was found to possess antioxidative, anti-inflammatory properties, and increased solubility; therefore, it can be applied to eliminate the progression of neurodegenerative diseases, such as AD. In addition, water modifications have advantages of probably being cost effective, easy to access, publicly acceptable, and generally safe. In this study, PAW was applied to young AD animals to explore its preventive effects on memory decline and the amyloid burden.

## Results

### Activity of scavenging free radicals by PAW

In experiments, the drinking water of AD mice was prepared every day using either fresh DI water or PAW. Thus, in the current experiments, we examined the ability of as-prepared and 1-day-aged PAW to scavenge free radicals compared to DI water to verify this ability. Figure S1 demonstrates the scavenging abilities of as-prepared and 1-day-aged PAW, compared to DI water, on active hydroxyl radicals. The four electro-spin resonance (ESR) splitting signals shown in Fig. S1A are characteristic of hydroxyl radicals^16^. Figure S1B shows the corresponding statistically significant results. Compared to DI water, the intensities of free radicals decreased by 36% (*p* = 0.0078) and 23% (*p* = 0.0026) in magnitude, respectively, with as-prepared and 1-day-aged PAW. Similarly, the ability of as-prepared and 1-day-aged PAW to scavenge radicals also demonstrated a positive effect on decreasing the corresponding ESR intensities of DPPH stable free radicals, as shown in Fig. S2. Compared to DI water, the intensities of free radicals decreased by 12% (*p* = 0.0020) and 9% (*p* = 0.022) in magnitude, respectively, with as-prepared and one-day-aged PAW.

### Memory decline was rescued in APP/PS1 mice with PAW treatment

**Figure 1:**
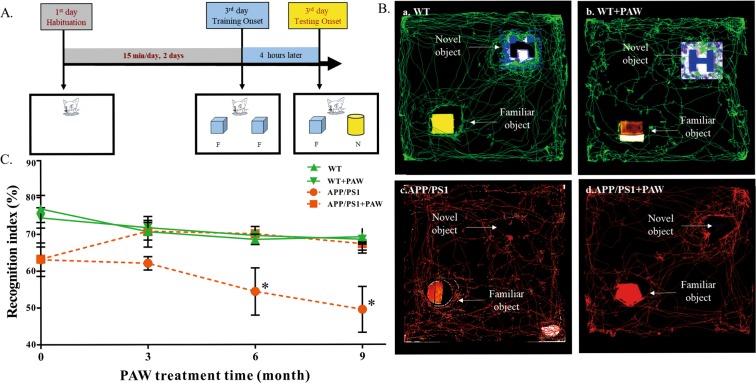
Memory function assessment by a novel object recognition (NOR) test at 0, 3, 6, and 9 months after plasmon-activated water (PAW) treatment beginning when animals were 5 months old. Scheme (**A**) shows the procedure for training and the test trails in this experiment. F, familiar object; N, novel object. (Ba–d) Tracks of four groups recorded with the Noldus Ethovision XT analytical system in the test phase of 9 months after treatment with PAW. WT, wild-type mice treated with deionized (DI) water (**B**-a), WT + PAW, wild-type mice treated with PAW (**B**-b), APP/PS1, APPswe/PSEN1dE9 mice treated with DI water (**B**-c), and APP/PS1 + PAW, APPswe/PSEN1dE9 mice treated with PAW (**B**-d). Statistical bars in the four groups (**C**) show the percentage of the recognition index. *p < 0.05 (two-way ANOVA), *n* = 5 or 6 in each group.

Memory decline is one of the clinical symptoms that occur in AD. To analyze the effects of PAW on memory decline in APP/PS1 mice, we conducted a novel object recognition (NOR) test every 3 months after treatment using 5-month-old APP/PS1 mice (Fig. 1A). Results of the NOR test did not differ between APP/PS1 mice fed PAW or no PAW (63.290% ± 3.284% vs. 63.123% ± 4.511%, *p* = 1.000) at the beginning. After 3 months of treatment, the performance of APP/PS1 mice given PAW had improved, but did not significantly differ from those mice without PAW (70.818% ± 2.402% vs. 64.200% ± 2.145%, *p* = 1.305). The recognition index of APP/PS1 mice without PAW had declined at 6 months (70.093 ± 1.153% vs. 56.260 ± 5.290%, *p* = 0.0178). After 9 months of treatment, the recognition index of APP/PS1 mice without PAW had significantly declined (67.354 ± 2.575% vs. 49.516 ± 6.216%, *p* = 0.0008). Wild-type (WT) mice with or without PAW treatment showed no difference from 3 to 9 months of treatment (Fig. 1B,C). These findings indicated that PAW treatment could prevent memory decline in APP/PS1 mice.

### ^18^F-Florbetapir animal positron emission tomography (PET)

**Figure 2:**
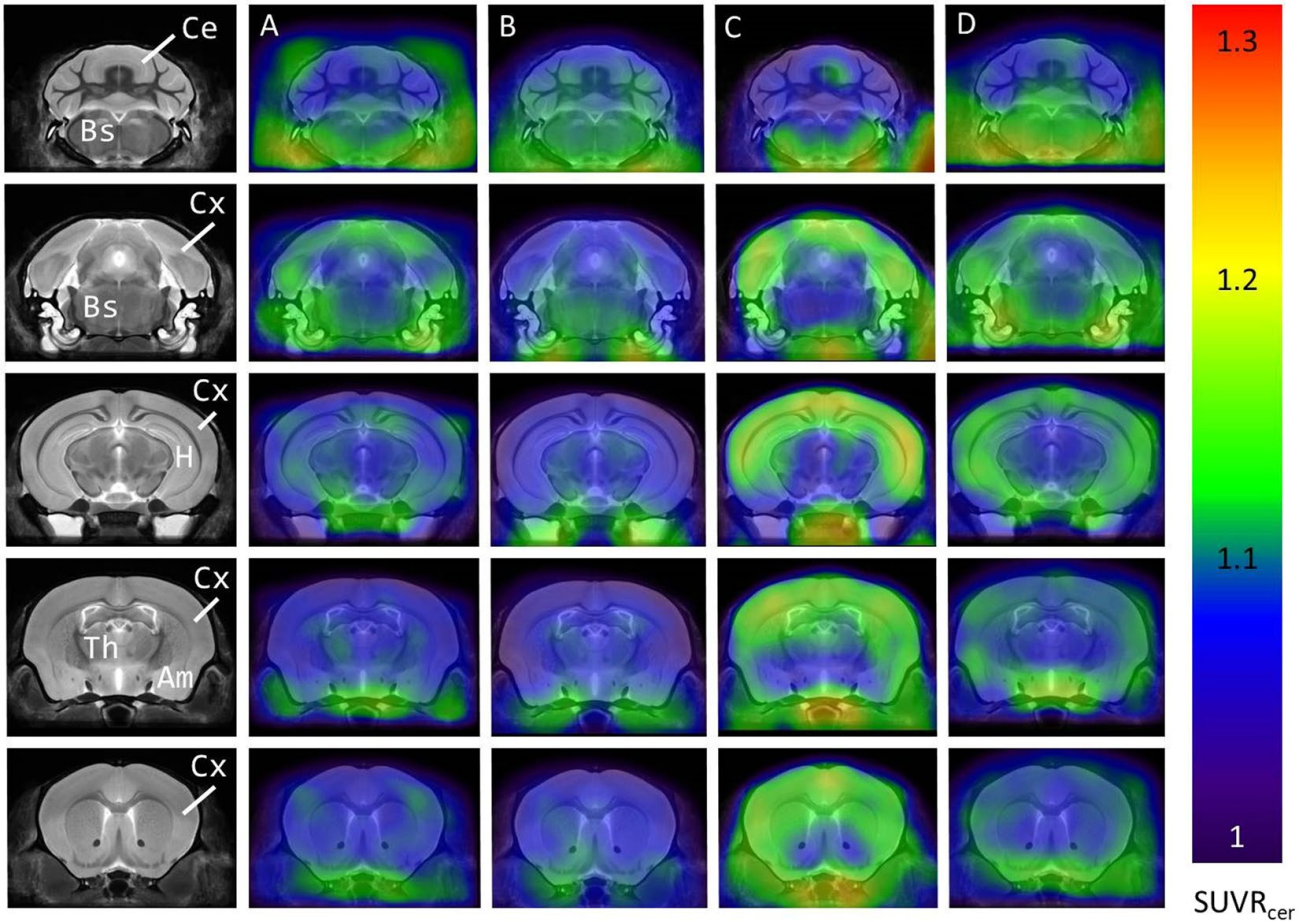
^18^F-Florbetapir animal positron emission tomographic (PET) images of 14~16-month-old APP/PS1 and age-matched wild-type mice. PET images were generated by averaging dynamic scan data at 30~60 min after an injection with the radiotracer, and these were overlaid onto magnetic resonance imaging (MRI) images. (Group A: wild-type mice without treatment; Group B: wild-type mice treated with plasmon-activated water (PAW); Group C: APP/PS1 mice without treatment; Group D: APP/PS1 mice treated with PAW).

**Figure 3:**
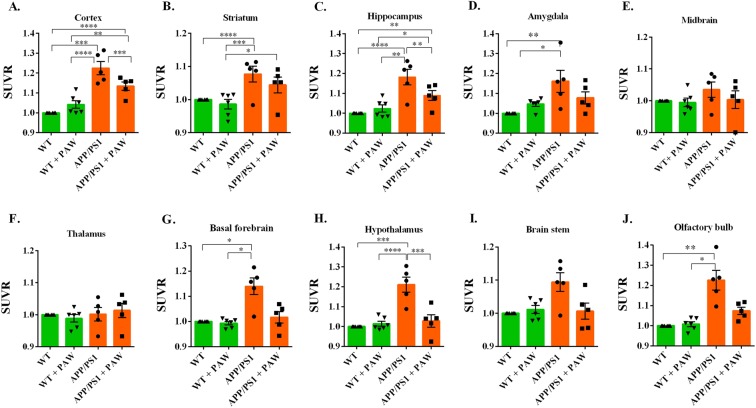
Comparison of relative 18F-florbetapir uptake levels in the cortex, striatum, hippocampus, amygdala, midbrain, thalamus, basal forebrain, hypothalamus, brain stem, and olfactory bulb (**A~J**) expressed as the region/cerebellum ratio for APP/PS1and wild-type mice with/without PAW treatment. The left line shows anatomical structures in the MRI/PET images. The right line shows the color bar of the standardized uptake value ratio (SUVR) compared to the cerebellum (SUVR_cer_). (*n* = 5 or 6 per group, by a repeated-measures ANOVA and post-hoc analysis, *p < 0.05, **p < 0.01, ***p < 0.001).

Representative average PET images (30~60 min after injection) of ^18^F-florbetapir in brains of APP/PS1 and WT mice with/without PAW treatment were prepared. Intense and diffusely increased uptake of radioactivity was observed in the cortex, striatum, hippocampus, amygdala, and brain stem regions of APP/PS1 mice (Fig. 2C); the radioactivity uptake was less intense in APP/PS1 mice undergoing PAW treatment (Fig. 2D). ^18^F-Florbetapir uptake in the abovementioned brain regions was lowest in WT C57BL/6 mice with/without PAW treatment (Fig. 2A,B). Of note, radioactivity uptake levels were similar in background regions of all animals, such as the cerebellum, midbrain, and thalamus. Taking into account all data, significant increases in the ^18^F-florbetapir uptake ratio were observed in the cortex (Fig. 3A), striatum (Fig. 3B), hippocampus (Fig. 3C), amygdala (Fig. 3D), basal forebrain (Fig. 3E), hypothalamus (Fig. 3H), and olfactory bulb (Fig. 3J) of APP/PS1 mice compared to WT mice. There were significantly reduced ^18^F-florbetapir uptake levels in the cortex (Fig. 3A), hippocampus (Fig. 3B), hippocampus (Fig. 3C), and hypothalamus (Fig. 3H) of APP/PS1 mice under PAW treatment compared to those without treatment. However, radioactivity uptake levels of the cortex and hippocampus in treated animals were still higher than those of control mice (Fig. 3). As expected, no significant difference in ^18^F-florbetapir uptake in any brain region was observed between control groups with or without treatment (i.e., WT vs. WT + PAW) (Table 1). These data suggest that PAW might reduce the formation of senile plaques in APP/PS1 mice.

**Table 1:** The standardized uptake value ratio (SUVR) in the cerebellum (SUVR_cer_) of each brain region.

Mean ± SEM	WT (n = 6)	WT + PAW (n = 6)	APP/PS1 (n = 5)	APP/PS1 + PAW (n = 5)
Cortex	0.926 ± 0.009	0.964 ± 0.009	1.127 ± 0.021	1.044 ± 0.018
Striatum	1.005 ± 0.012	0.991 ± 0.007	1.077 ± 0.013	1.045 ± 0.014
Hippocampus	1.002 ± 0.018	1.026 ± 0.008	1.175 ± 0.021	1.083 ± 0.014
Amygdala	0.966 ± 0.016	1.011 ± 0.017	1.113 ± 0.037	1.035 ± 0.018
Mibrain	1.042 ± 0.014	1.036 ± 0.011	1.076 ± 0.014	1.043 ± 0.018
Thalamus	1.027 ± 0.010	1.015 ± 0.009	1.025 ± 0.014	1.037 ± 0.015

### Amyloid plaque and p-tau burden decreased in the hippocampus in APP/PS1 mice undergoing PAW treatment

**Figure 4:**
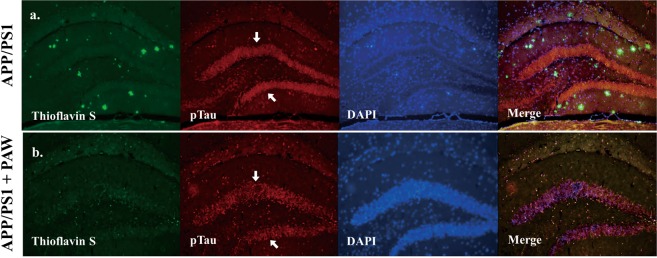
Detection of the amyloid beta and phosphorylated (p)-tau burden in the hippocampus of APP/PS1 mice by immunocytochemical staining. (**A**) APP/PS1 mice, (**B**) APP/PS1 mice treated with plasmon-activated water (PAW). Triple staining was performed using 4% paraformaldehyde fixation. Thioflavin-S was used to detect amyloid plaque. Protein levels of p-tau were detected by an AT8 monoclonal antibody and Alexa 594-conjugated secondary antibody. The green channel represents thioflavin-S staining, the red one represents p-tau, and the blue channel represents DAPI staining.

**Figure 5:**
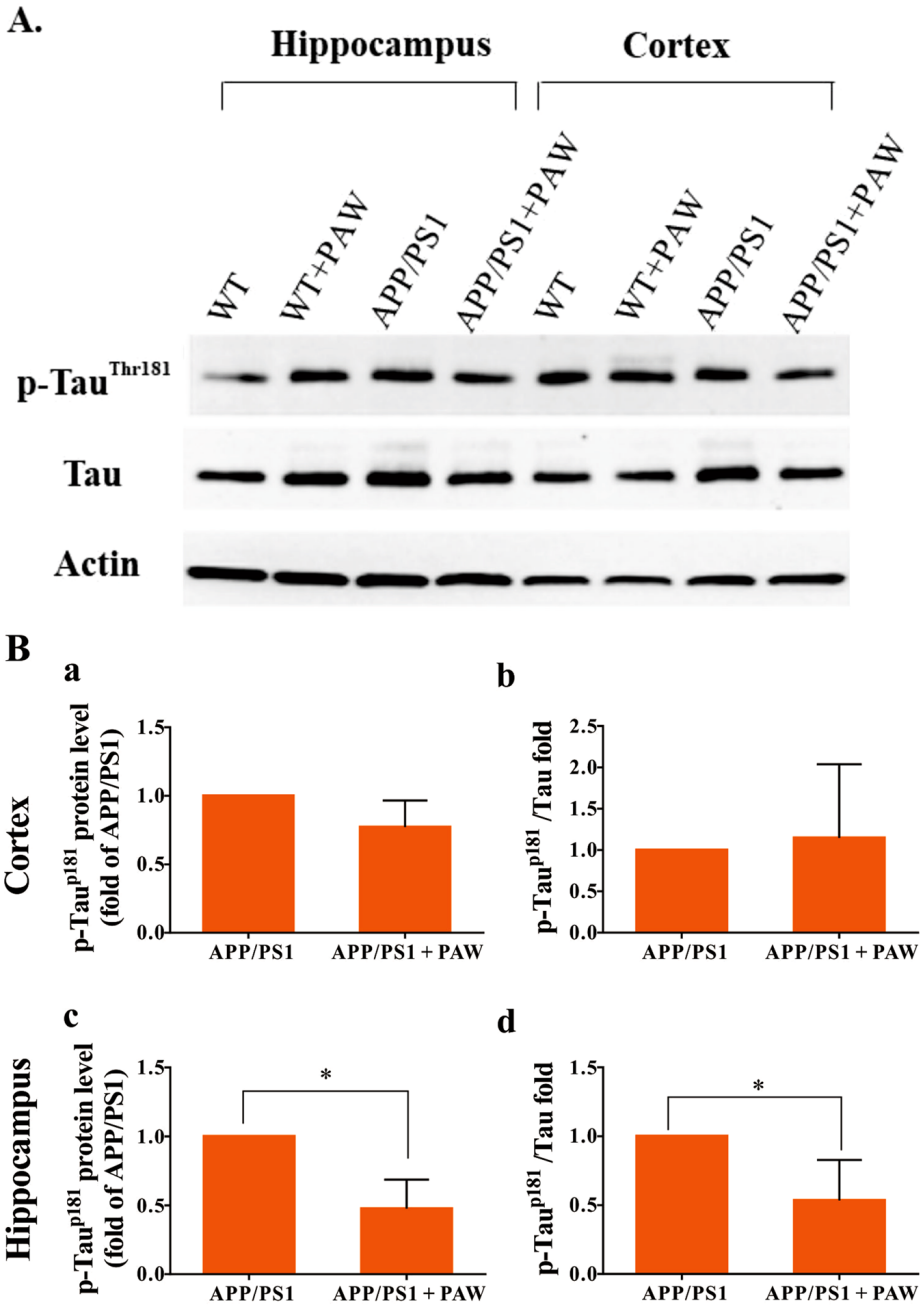
Measurement of phosphorylated (p)-tau in APP/PS1 mice treated with plasmon-activated water (PAW). Total tau and p-tau were measured by Western blotting (**A**). Results from Western blotting were statistically analyzed (**B**). WT, wild-type mice treated with deionized (DI) water; WT + PAW, wild-type mice treated with PAW; APP/PS1, APP/PS1 mice treated with DI water; and APP/PS1 + PAW, APP/PS1 mice treated with PAW. *n* = 4 in each group.

The amyloid plaques and p-tau burden in the hippocampus was reported to be a biomarker of AD. To analyze the effects of PAW on the amyloid plaques and p-tau burden, we conducted thioflavin-S staining and immunocytochemistry in APP/PS1 mice at 9 months after treatment. The amyloid plaques and p-tau level had increased at APP/PS1 mice (Fig. 4). Compared to APP/PS1 mice, the p-tau level in APP/PS1 mice treated with PAW had significantly declined (Fig. S3). We further analyzed protein levels of p-tau in the cortex and hippocampus of APP/PS1 mice with or without PAW treatment for 9 months by Western blotting (Fig. 5A). The protein level of p-tau had significantly decreased in the hippocampus but not in the cortex of APP/PS1 mice treated with PAW compared to untreated mice (Fig. 5Ba,c). The ratio of p-tau/tau had also decreased in the hippocampus but not in the cortex (Fig. 5Bb,d). These data suggest that PAW might reduce the p-tau burden in the hippocampus of APP/PS1 mice.

### Oligomeric amyloid level was not affected in APP/PS1 mice treated with PAW

**Figure 6:**
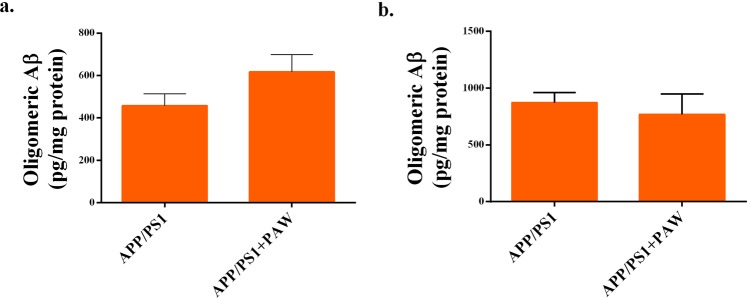
Quantification of oligomeric amyloid-β (Aβ) in APP/PS1 mice treated with plasmon-activated water (PAW). Protein levels of oligomeric Aβ in the hippocampus (**A**) and cortex (**B**) of APP/PS1 or WT mice treated with deionized (DI) water or PAW were measured by an ELISA. WT, wild-type mice treated with DI water; WT + PAW, wild-type mice treated with PAW; APP/PS1, APP/PS1 mice treated with DI water; and APP/PS1 + PAW, APP/PS1 mice treated with PAW. *n* = 4 in each group.

**Figure 7:**
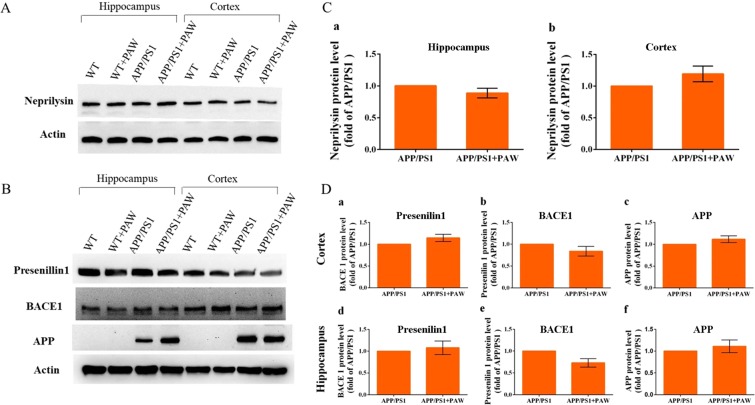
Measurement of neprilysin, presenilin 1, BACE1, and the amyloid precursor protein (APP) in APP/PS1 mice treated with plasmon-activated water (PAW). (**A**) Neprilysin was measured by Western blotting with actin as an internal control. (**B**) Presenillin1, BACE1, and the APP were measured by Western blotting with actin as an internal control. **C**-a~b show the statistical data of neprilysin in the cortex (a) and hippocampus (b). **D**-a~c show protein levels of BACE1 (a), presenilin 1 (b), and the APP (c) in the cortex. **B**-d~f show protein levels of BACE1 (d), presenilin 1 (e), and the APP (f) in the hippocampus. WT, wild-type mice treated with deionized (DI) water; WT + PAW, wild-type mice treated with PAW; APP/PS1, APPswe/PSEN1dE9 mice treated with DI water; and APP/PS1 + PAW, APP/PS1 mice treated with PAW. n = 4 in each group.

To analyze the effect of the oligomeric amyloid level on APP/PS1 mice treated with PAW, we prepared an ELISA experiment. Oligomeric amyloid levels in the hippocampus (458.2 ± 55.31 pg/mg vs. 616.9 ± 81.73 pg/mg, *p* = 0.622) (Fig. 6A) and cortex (873.2 ± 87.5 pg/mg vs. 768.9 ± 181 pg/mg, *p* = 0.538) (Fig. 6B) of APP/PS1 mice showed no difference between mice with or those without PAW treatment. Further analyses of protein levels of neprilysin (Fig. 7A,C), PSEN1, BACE1, and APP (Fig. 7B,D) in APP/PS1 and WT mice showed that protein levels did not differ. Collectively, results suggested that the effect of PAW on APP/PS1 mice was not at the protein level of oligomeric amyloid.

## Discussion

The real cause of AD is still unclear. Aβ containing 40 or 42 amino acids is the main component of extracellular senile plaques, which are the most important marker of AD pathology. There is compelling evidence supporting the crucial role of amyloid in the pathophysiology of AD, from genetic, *in vitro*, and *in vivo* studies^9^. Transgenic animals, like APPswe/PSEN1dE9 (APP/PS1) mice, reveal early and progressive accumulation of amyloid and develop memory decline, similar to symptoms in humans, from about 3 months old^22^. The amyloid PET scan has become the most important biomarker tool for diagnosing AD. However, the burden of amyloid in the brain is not perfectly correlated with the severity of AD, and almost all clinical trials of amyloid-cleaning therapy have failed^7,14^. Most studies showed that although the amyloid burden was reduced, dementic symptoms continued to progress^23^. The amyloid hypothesis was recently challenged^24^. To test the amyloid hypothesis, treatment in many ongoing trials was moved to the early or asymptomatic phase, to try and prevent mental decline^25^. In this study, we tested the effects of PAW on transgenic APP/PS1 mice. Based on prior research, significant amyloid accumulation appeared at 3 months of age, behavior and memory declines occurred a few months later, and abnormal amyloid PET results were found after 9 months of age in APP/PS1 animals^26^. Therefore, AD mice were given PAW from 5 months old, and memory surveys with the NOR test were conducted every 3 months. Results showed that memory continuously and obviously declined in AD mice without PAW, but memory decline was slower in AD mice treated with PAW. The memory performance became significantly different at 6 months after treatment (Fig. 1). Amyloid PET scans showed that the amyloid burden was higher in AD mice without PAW treatment, especially in the cortex, hippocampus, and hypothalamus (Figs 2 and 3). These findings support the close relationships of amyloid burdens in the cortex and hippocampus with memory declines. Amyloid-reduction therapy should potentially be effective. Results also implied that treatment to reduce the amyloid burden might begin in the early stage, before or soon after the accelerating accumulation of amyloid. PAW has been tested in a few diseases, and its antioxidative and anti-inflammatory effects were documented^19,20^. The mechanisms of its reduction of the amyloid burden by PAW could be in multiple manners. The antioxidative and anti-inflammatory actions could suppress APP, β-secretase, and γ-secretase gene expressions and reduce the Aβ formation^27–29^. Inhibition of polymerization from monomeric or oilgomeric Aβ is also a possible mechanism^30^. In our previous report, the amount of small water clusters created in PAW showed a slightly negative charged^20^. These small water clusters might prevent polymerization of Aβ in APP/PS1 mice. Enhancement of expressions of amyloid-degradation enzymes, including neprilysin, is another possible mechanism^31^. In this study, oligomeric Aβ protein levels were measured, but they showed no change with PAW treatment in APP/PS1 mice (Fig. 6). The synthesis of the Aβ protein is regulated by neprilysin, PSEN1, BACE1, and the APP. Levels of these proteins also did not change in our present data. We suggest that the effect of PAW on reducing the formation of senile plaques did not occur in the synthesis of the Aβ protein, but in preventing aggregations of Aβ proteins. Further studies on the reduction mechanism of PAW are needed.

The tau protein which is also the another main component of pathological biomarker, intracellular neurofibrillary tangles, is considered to be another target for the diagnosis and treatment of AD^14^. Interactions between amyloid and tau are well studied^22^. Amyloid enhances tau phosphorylation, and amyloid alters tau splicing^32,33^. In this study, p-tau accumulated in the hippocampus of APP/PS1 mice with a high amyloid burden along with aging. APP/PS1 mice given PAW showed reduced p-tau deposition in the hippocampus compared to APP/PS1 mice without PAW, and such deposition is considered the beginning site of AD pathology, by comparison of APP/PS1 mice without PAW (Fig. 4). These findings suggest that PAW can reduce p-tau accumulation, which might be attributed to reduced amyloid plaque formation in APP/PS1 mice. However, the detailed mechanisms still need further investigation.

Inflammation occurs in the pathologically vulnerable region of AD brains and contributes to AD pathogenesis. Interleukin (IL)-1 and IL-6 are immunoregulatory cytokines which are overexpressed in the AD brains^34^. Reduction of inflammation in AD brain might help to delay the progression of AD. In our present data, we analyzed the inflammation by measuring protein levels of IL-1β and IL-6 using an ELISA assay. Although the protein levels of IL-1β and IL-6 were not significantly reduced in PAW-treated APP/PS1 mice, decreased values were observed (Fig. S4). The number of mice used for the analysis of inflammation was not sufficient to show a significant difference. Further experiments are needed to elucidate the reduction effects on inflammation in APP/PS1 mice.

This study showed that PAW reduced the amyloid and p-tau burden in APP/PS1 mice. Although the precise molecular mechanisms are still unclear, our data might provide a new potential strategy for preventing of AD. Although there might be multiple mechanisms, aggregation or polymerization of amyloid, p-tau accumulation and anti-inflammation could all be involved. In conclusion, PAW confers effects of reducing amyloid and p-tau accumulation, borderline significant anti-inflammation and decelerating memory declines. These novel findings indicate the potential use of PAW in the therapy of early AD.

## Methods

### Animals

APPswe/PSEN1dE9 (APP/PS1) transgenic mice were provided by Dr. Rita P‐Y Chen of the Institute of Biological Chemistry, Academia Sinica (Taipei, Taiwan)^35^. The genotype of the transgenic mice was analyzed by a polymerase chain reaction (PCR) with Hot-NaOH-extracted genomic DNA from the tail. We used 5-month-old male APP/PS1 mice in this study and their littermates as controls. The mice were individually kept in ventilated cages with feeding racks and water bottles were attached to the front panel of the cage, which allowed animals ad libitum access to food and water. Mice were maintained on a 12-h light/dark cycle (12L/12D; lights on at 07:00 and lights off at 19:00). The food and deionized (DI) water or PAW were freely available, and the water was changed daily. Six (APP/PS1) mice for each group were fed with PAW or DI water for 9 months, beginning from the age of 5-months. All the experiments were approved by the Experimental Animal Review Committee of Taipei Medical University, and all methods were performed in accordance with guidelines of the National Institute of Health, Taiwan.

Full Methods and any associated references are available in the online version of the paper.

## Supplementary information

Plasmon-Activated Water Reduces Amyloid Burden and Improves Memory in Animals with Alzheimer’s Disease
