# Supplementary material for: Plasmon-Activated Water Reduces Amyloid Burden and Improves Memory in Animals with Alzheimer’s Disease
Source: Sci Rep. 2019 Sep 13;9:13252. doi: 10.1038/s41598-019-49731-8 (PMC6744477; doi:10.1038/s41598-019-49731-8)
Supplement: Supplementary file 1 — Plasmon-Activated Water Reduces Amyloid Burden and Improves Memory in Animals with Alzheimer’s Disease [file 41598_2019_49731_MOESM1_ESM.pdf]

Supplementary Information:

Plasmon-Activated Water Reduces Amyloid Burden and Improves Memory in Animals with Alzheimer's Disease

Chia-Hsiung Cheng[1], Kun-Ju Lin[2,3], Chien-Tai Hong[4,5], Dean Wu[4,5], Hung-Ming Chang[6], Cheng-Huan Liu[7], Ing-Tsung Hsiao[2,3], Chih-Ping Yang[1], Yu-Chuan Liu[1] and Chaur-Jong Hu[4,5,8]

[1]Department of Biochemistry and Molecular Cell Biology, School of Medicine, College of Medicine, Taipei Medical University, 250 Wuxing St., Taipei 11031, Taiwan

[2]Department of Nuclear Medicine and Molecular Imaging Center, Linkou Chang Gung Memorial Hospital, 5 Fuxing St., Tao-Yuan, Taiwan

[3]Healthy Aging Research Center and Department of Medical Imaging and Radiological Sciences, College of Medicine, Chang Gung University, 259 Wenhua 1st Rd., Taoyuan City 33302, Taiwan

[4]Department of Neurology, School of Medicine, College of Medicine, Taipei Medical University, 250 Wuxing St., Taipei 11031, Taiwan

[5]Department of Neurology and Dementia Center, Shuang Ho Hospital, Taipei Medical University, 291 Jhongjheng Rd., Jhonghe, New Taipei City 23561, Taiwan

[6]Department of Anatomy and Cell Biology, School of Medicine, College of Medicine, Taipei Medical University, 250 Wuxing St., Taipei 11031, Taiwan

[7]Science Department of Physiology, McGill University, 3655 Promenade Sir William Osler, Montreal, Quebec, Quebec H3G 1Y6, Canada

[8] PhD Program of Neural Regenerative Medicine, College of Medical Science and Technology, Taipei Medical University, 250 Wuxing St., Taipei 11031, Taiwan

C.-H.C. and K.-J.L. contributed equally to this work.

Correspondence and requests for materials should be addressed to C.-J.H. (email: chaurjongh@tmu.edu.tw) and Y.-C.L. (email: liuyc@tmu.edu.tw)

Preparation of plasmon-activated water (PAW). The PAW preparation conditions were reported previously.[1] Typically, to prepare PAW, deionized (DI) water (pH 7.23, temperature = 23.5 °C) was passed through a glass tube filled with Au NPs-adsorbed ceramic particles under illumination with green light-emitting diodes (LEDs, with wavelength maxima centered at 530 nm). Then the PAW (pH 7.25, temperature = 23.3 °C) was collected in glass sample bottles for subsequent use as soon as possible. To examine the purity of the prepared PAW further inductively coupled plasma-mass spectrometric (ICP-MS) analyses indicated that the concentrations of the slightly dissolved metals in the PAW were ca. 0.62 ppb for Au, 43 ppb for Na, 25 ppb for K, 23 ppb for Al, 13 ppb for Mg, 4.5 ppb for Ca and 0.41 ppb for Fe, respectively. Excluding Au, the total equivalent molar concentration of these dissolved metals is equal to ca. 6.9×10^{-6} N. This measured value was ca. 2.4×10^{-7} N for DI water as a reference. Also, the slightly dissolved Au and the total equivalent molar concentration of other dissolved metals in PAW (light-free) are 0.57 ppb and 5.2×10^{-6} N, respectively. Mice were treated with PAW or DI water for 9 months beginning from the age of 5 months to 14 months.

Novel object recognition (NOR) test. The test was performed in a black plastic and black box (45 ×45×60 cm) with a camera on the top for behavior recording at 0, 3, 6, and 9 months of PAW treatment. The procedure included three phases: habituation, training, and testing. On day 1 to day 3 (habituation), a mouse was placed in the box for 15 min and allowed to freely explore the environment. On day 3, after habituation, each mouse was received 15 min of training in the box with two identical objects, and then it was returned to its home cage. Following a 4-h delay, the mouse was placed back into the box where it was presented one familiar object and one novel object for 15 min. Objects and the test area were cleaned with 70% ethanol after each task. The video was analyzed by Noldus software (Noldus, Leesburg, VA, USA). The amount of time spent on the novel object was compared to the time spent on the familiar object. Novel object preference scores were calculated as the percentage of total time spent on the novel object : NOR %= [(time of novel object) / (time of novel object) + (time of familiar object)] × 100). Statistical analysis of each groups was performed by a two-way analysis of variance (ANOVA) with a post-hoc analysis (* $p < 0.05$, ** $p < 0.01$, and *** $p < 0.001$) where measurements were summarized as the mean ± standard error of the mean (SEM) for each group.

^{18}F-Florbetapir positron emission tomography (PET) imaging. The radiosynthesis of ^{18}F-florbetapir (AV-45/Amyvid)[2] and amyloid PET data acquisition[3] were previously described by our group. Animal PET studies were conducted in 14-month-old APP/PS1

and age-matched C57BL/6 mice (n=5 or 6 per treatment group). All PET images were acquired using a preclinical Inveon PET system (Siemens Medical Solutions, Knoxville, TN, USA). Each mouse was subjected to a 60-min dynamic scan under isoflurane anesthesia (1.5% in oxygen gas) after receiving a single bolus injection of ^{18}F-florbetapir (19.52 ± 1.06 MBq in 0.1 mL saline) through a tail vein.

All image data were processed and analyzed using PMOD image analysis software (vers. 3.7, PMOD Technologies Ltd, Zurich, Switzerland). To ensure proper volume of interest (VOI) placement on PET images, we manually coregistered in each mouse PET images with the PMOD built-in T1-weighted magnetic resonance imaging (MRI) template. The built-in volume of interests (VOIs) of the cortex, striatum, hippocampus, amygdala, thalamus, mid brain, and cerebellum from the MRI template were applied to the fused PET images for quantification. Mean ^{18}F-florbetapir activities, corrected for radioactive decay, were evaluated for each VOI on integrated PET images recorded over the 30~60 minutes acquisition period. ^{18}F-Florbetapir standardized uptake values of each VOI were calculated by dividing the mean radioactivity counts by the injected dose and body weight. The cortical standardized uptake value (SUV) ratios (SUVRs) were calculated as $SUV_{cortical}$/ $SUV_{cerebellum}$, where the cerebellum was used as the reference region.

Enzyme-linked immunosorbent assay (ELISA) for oligomeric amyloid. The frozen cortical and hippocampal tissue samples were homogenized in 1 mL tapered tissue grinders in 500 µL of ice-cold TBS buffer (50 mM Tris, 150 mM NaCl, and 2 mM EDTA, at pH 7.4) with a protease inhibitor cocktail (Sigma, St. Louis, MO, USA). The homogenates were transferred to 1.5-mL of tubes and centrifuged at 20,000 g and 4 °C for 20 min. The final supernatant was quantified using the Bradford protein assay (Bio-Rad #500-0006) and diluted 500-fold before ELISA quantification using human amyloid beta oligomers assay kit (IBL, Fujioka, Japan) and cytokines detection kits for assessing IL-6 and IL-1β (DY401 and DY406, R&D, Minneapolis, MN, USA) according to the manufacturer's protocol. The absorbance was measured at 450 nm with a SpectraMax microplate reader (Molecular Devices, San Jose, CA, USA). All samples were analyzed in duplicate.

Western blotting analysis. The frozen cortical and hippocampal tissue samples were homogenized in 1 mL of tapered tissue grinders in 8-fold of ice-cold TBS buffer (50 mM Tris, 150 mM NaCl, and 2 mM EDTA, at pH 7.4) with a protease inhibitor cocktail (Sigma) and a phosphatase inhibitor (BioShop, Ontarrio, Canada). After quantifying the protein content using the Bradford protein assay (Bio-Rad, CA, USA), 20µg of protein was resolved in an 10% EVO gel (GeneDirex, Miaoli City, Taiwan) by

sodium dodecylsulfate polyacrylamide gel electrophoresis (SDS-PAGE) and transferred to a polyvinylidene difluoride (PVDF) membrane (Pall, New Port Richey, FL, USA). The membrane was blocked using PBS containing 5% bovine serum albumin (BSA), which was followed by incubation with a primary antibody overnight at 4 °C. After washing with PBST containing 0.25% Tween-20, the membranes were treated with horseradish peroxidase (HRP)-conjugated secondary antibodies. Anti-p-tau (GTX50171) and anti-tau (GTX50451) antibodies were purchased from GeneTex (GeneTex, Hsinchu City, Taiwan). The anti-presenilin 1 antibody was purchased from Santa Cruze (Santa Cruze, Dallas, TX, USA). Anti-actin was purchased from Millipore (Billerica, MA, USA). Anti-neprilysin and anti-BACE-1 antibodies were purchased from R&D (R&D, MN, USA). The anti-APP antibody was purchased from BioLegend (BioLengend, San Diego, CA, USA). Goat anti-mouse-immunoglobulin G (IgG), anti-rat-IgG and anti-rabbit-IgG antibodies were purchased from Santa Cruz (Santa Cruz, CA, USA). The protein bands were visualized using the Western Lightning Enhanced Chemiluminescence Substrate kit (Perkin Elmer, Waltham, MA, USA). Blot images were digitized by the BioSpectrum AC Imaging System (UVP, Upland, CA, USA), and bands were quantified by the UVP Vision Works software.

Phospho-Tau immunocytochemistry and thioflavin-S staining

Mice were anesthetized with isoflurane (2 %) and the animals were taken cardiac-perfused with 4 % paraformaldehyde. Tissues were incubated at 4 °C for post-fixation overnight. A brain was cut into 5-um slices. Parafilm slides were deparafflinzed and dehydrated with Nova Histo (BIONOVAS, Toronto, Canada) in a microwave for 3 min, and washed with tap water for several times. Brain slides were blocked with PBS containing 0.5 % Triton X-100 (vol/vol) and 5 % BSA. After rinsing, the primary p-tau antibody (AT8, ThermoFisher, Waltham, MA, USA) was incubated overnight. After washing, an Alexa 594-conjugated secondary antibody was incubated for 1 h. For thioflavin-S staining, tissue slides were continuously placed in a 1% thioflavin-S solution (Sigma-Aldrich, St. Louis, MO, USA) for 10 min, and then differentiated in 70 % ethanol. Images were taken with the EVOS FL Imaging System (ThermoFisher).

Measurement of free radicals by electron spin resonance (ESR) spectroscopy. For ESR measurements, a Bruker EMX ESR spectrometer was employed. ESR spectra were recorded at room temperature using a quartz flat cell designed for solutions. The dead times between sample preparation and ESR analysis were exactly 1.5 and 10 min for experiments of hydroxyl and DPPH free radicals, respectively, after the last addition. Conditions of ESR spectrometry were as follows: 20 mW power at 9.78 GHz, with a scan range of 100 G and a receiver gain of 6.32×10^4.

Sample preparation for measuring hydroxyl free radicals. Hydroxyl free radicals were obtained by using the well-known Fenton reaction, in which ferrous iron donates an electron to hydrogen peroxide to produce the hydroxyl free radical.[4,5] Because the produced hydroxyl free radicals are very unstable, they are captured by spin-trapping using DMPO to form more-stable complex radicals for exact detection. The sample preparation is described here. First, 140 μL DI water or PAW was added to a microtube (Eppendorf, Hauppauge, NY, USA). Then 20 μL PBS (10x) was added to the tube. A complex of EDTA-chelated iron(II) was prepared by mixing 0.5 mM iron(II) chloride tetrahydrate and 0.5 mM EDTA of equal volumes. Subsequently, 20 μL EDTA-chelated iron(II) (0.25 mM), 10 μL H_2O_2 (0.2 mM) and 10 μL DMPO (2 M) were sequentially added to the tube. The final volume in the tube was 200 μL. Exactly 1.5 min after from the addition of DMPO, the ESR analysis was performed. To obtain an ESR spectrum, a sample was scanned for ca. 1.5 min, accumulated eight times, and all signals were averaged.

Sample preparation for measuring DPPH free radicals. Compared to hydroxyl free radicals DPPH is a kind of stable free radicals. Sample preparation is described here. DPPH was dissolved in methanol to prepare a 4 mM DPPH solution. Then the prepared DPPH solution and DI water (or PAW) were mixed (100 μL each) in a microtube. The final concentration of DPPH in the solution was 2 mM. Exactly 10 min after mixing DPPH and water, the ESR analysis was performed. When measuring an ESR spectrum, a sample was scanned for one time (ca. 42 s).

References

1. Chen, P. T. *et al.* Polyhydroxycurcuminoids but not curcumin upregulate neprilysin and can be applied to the prevention of Alzheimer's disease. *Sci Rep.* **6**, 29760-29771 (2016).
2. Yao, C. H. *et al.* GMP-compliant automated synthesis of [(18)F]AV-45 (florbetapir F 18) for imaging beta-amyloid plaques in human brain. *Appl Radiat Isot* **68**, 2293-2297 (2010).
3. Lin, K. J. *et al.* Whole-body biodistribution and brain PET imaging with [18F]AV-45, A novel amyloid imaging agent--A pilot study. *Nucl Med Biol* **37**, 497-508 (2010).
4. Ohsawa, I. *et al.* Hydrogen acts as a therapeutic antioxidant by selectively

reducing cytotoxic oxygen radicals. *Nat. Med*. **13**, 688-694 (2007).

5. Liu, Y. *et al.* Cell death from antibiotics without the involvement of reactive oxygen species. *Science* **339**, 1210-1213 (2013).

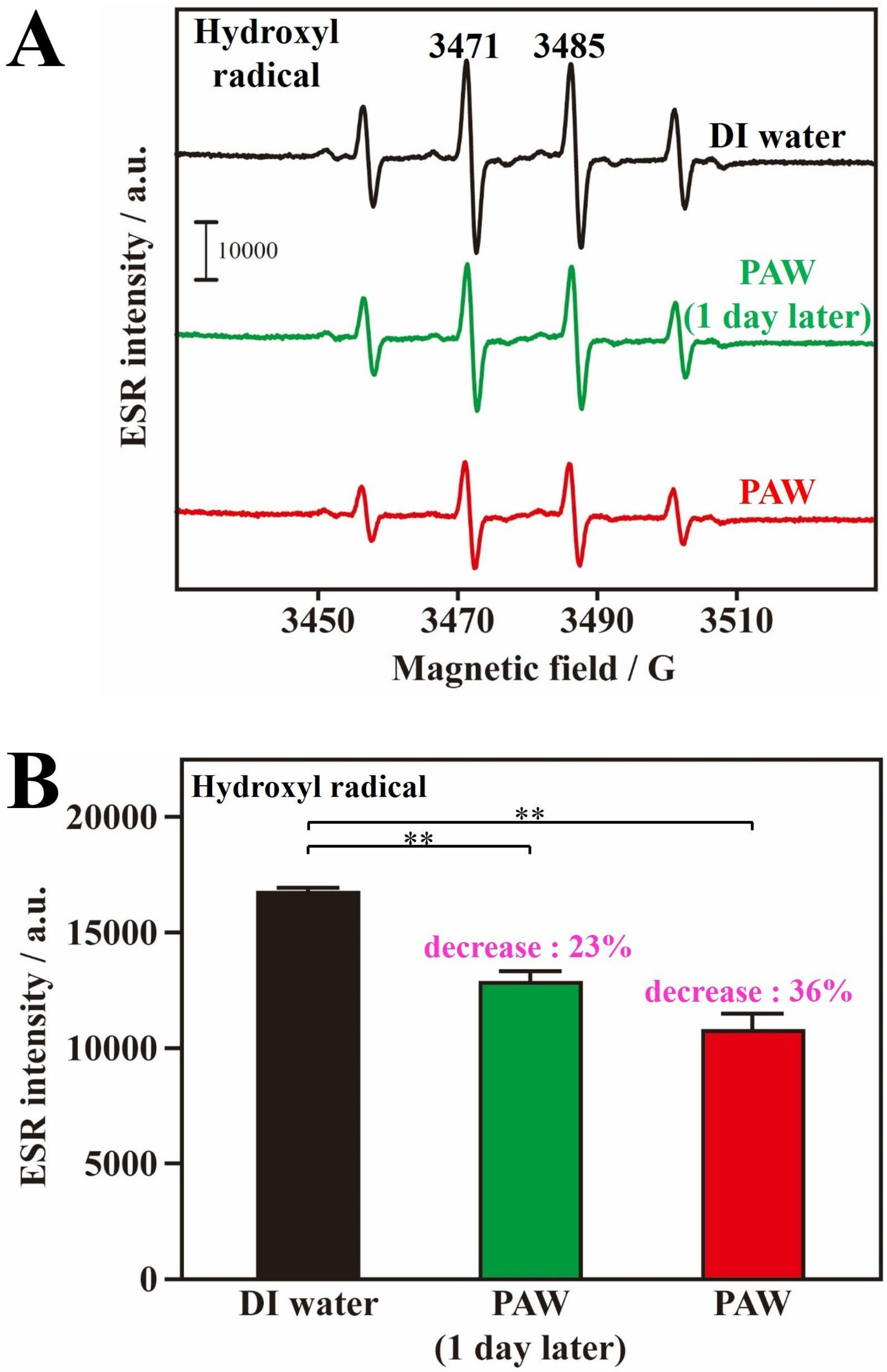

Figure S1. ESR spectra of hydroxyl free radicals based on as-prepared plasmon-activated water (PAW) (red), one-day-aged PAW (green), and deionized (DI) water (black) for reference. (A) Original spectra. (B) Statistical results. **, $p<0.01$.

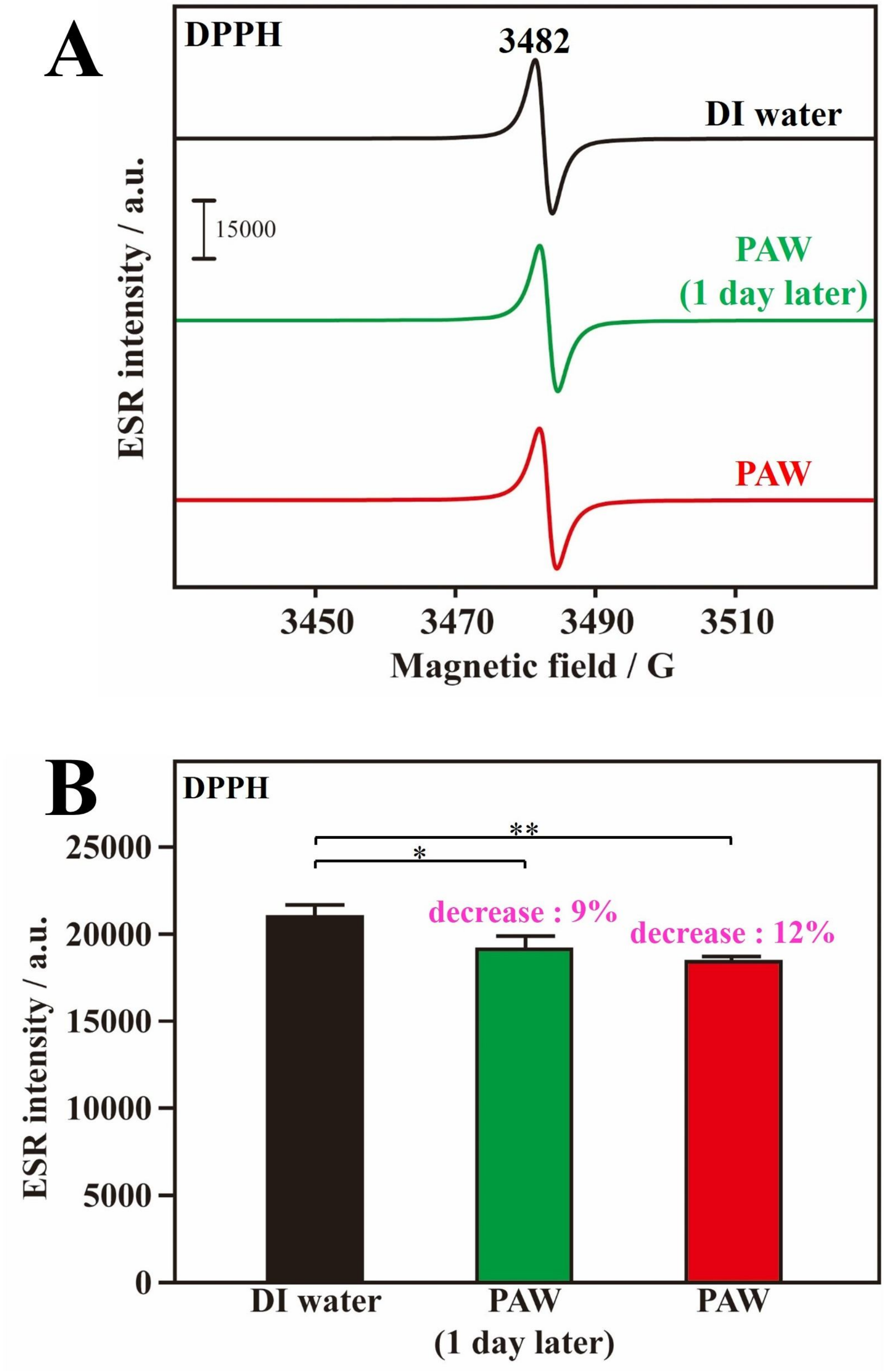

Figure S2. ESR spectra of DPPH free radicals based on as-prepared plasmon-activated water (PAW) (red), one-day-aged PAW (green) and deionized (DI) water (black) for reference. (A) Original spectra. (B) Statistical results. *, $p<0.05$; **, $p<0.01$.

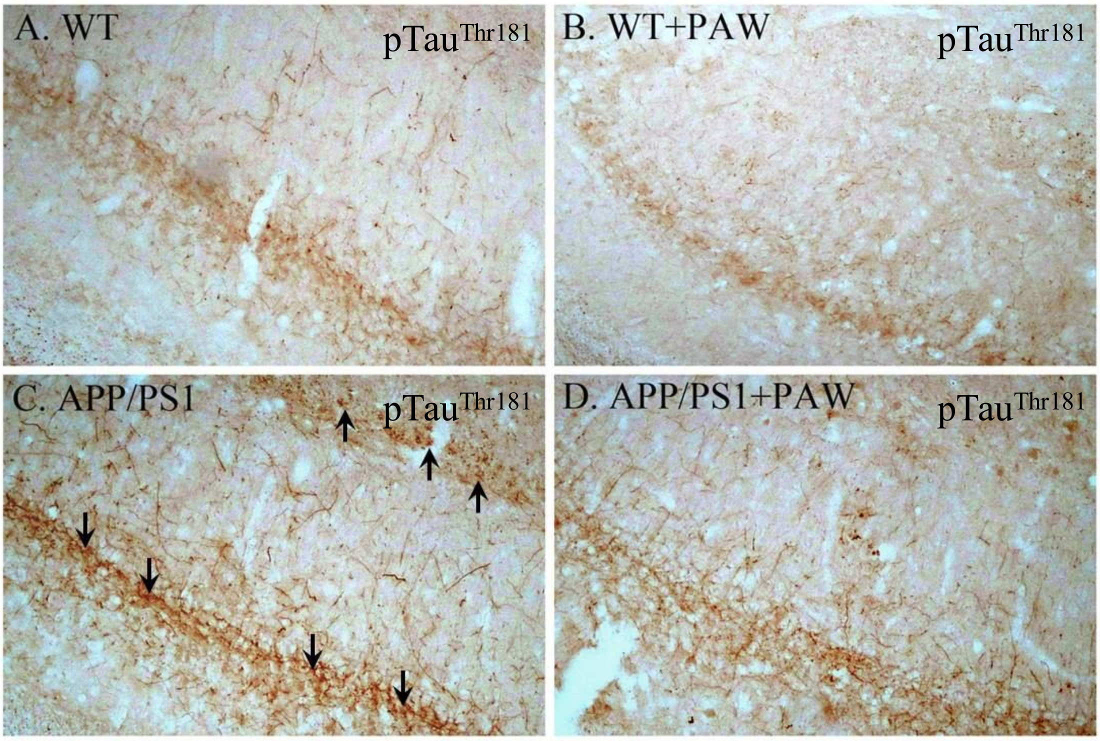

Figure S3. Detection of phosphorylated (p)-tau burden in the hippocampus of APP/PS1 mice by IHC. (A) Wild-type mice, (B) wild-type mice treated with plasmon-activated water (PAW), (C) APP/PS1 mice, (D) APP/PS1 mice treated with PAW. Untreated, wild-type (WT); AD, APP/PS1 mice.

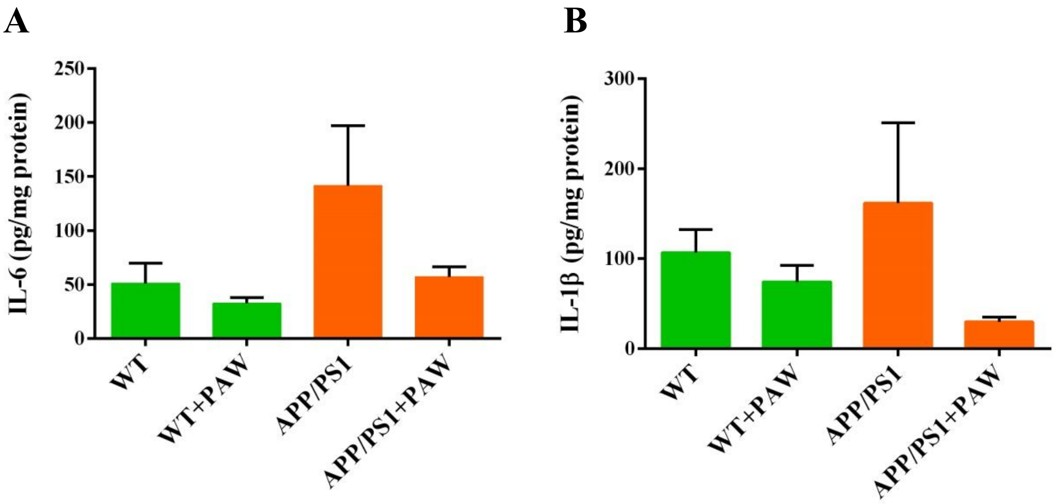

Figure S4. Quantification of interleukin (L-)6 and IL-1β in the hippocampus by ELISA experiments. Protein levels of IL-6 (a) and IL-1β (b) were measured in wild-type (WT) mice, WT mice treated with plasmon-activated water (PAW), APP/PS1 mice, and APP/PS1 mice treated with PAW. *n*=4 in each group.

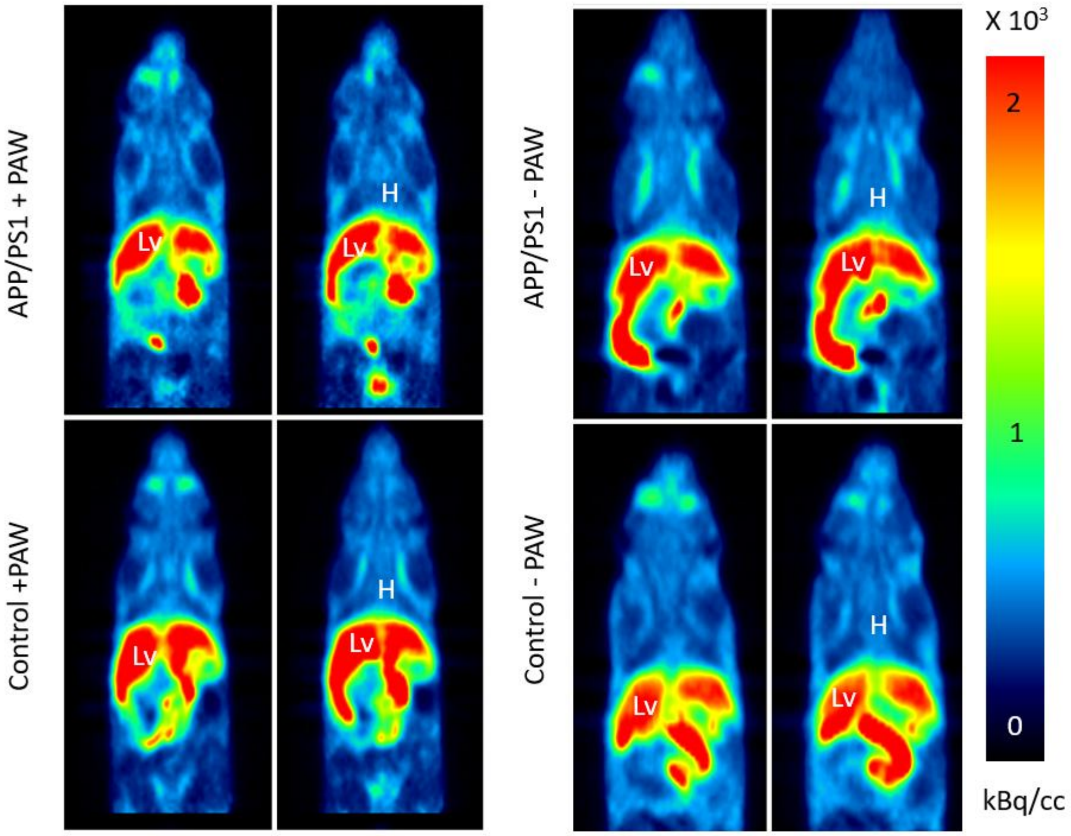

Figure S5. Dynamic whole body 18F-florbetapir PET images of APP/PS1 and control animals with/without plasmon-activated water (PAW) treatment. (H, heart; Lv, liver).

Table S1. The standardized uptake value ratio (SUVR) to the cerebellum ($SUVR_{cer}$) of each brain region.

Mean ± SEM	WT (n=6)	WT+PAW (n=6)	APP/PS1 (n=5)	APP/PS1+PAW (n=5)
Cortex	0.926±0.009	0.964±0.009	1.127±0.021	1.044±0.018
Striatum	1.005±0.012	0.991±0.007	1.077±0.013	1.045±0.014
Hippocampus	1.002±0.018	1.026±0.008	1.175±0.021	1.083±0.014
Amygdala	0.966±0.016	1.011±0.017	1.113±0.037	1.035±0.018
Midbrain	1.042±0.014	1.036±0.011	1.076±0.014	1.043±0.018
Thalamus	1.027±0.010	1.015±0.009	1.025±0.014	1.037±0.015

SEM, standard error of the mean; WT, wild-type mice; PAW, plasmon-activated water; APP/PS1, APPswe/PS1dE9 transgenic mice.
